# The Effect of Early Time-Restricted Feeding on Glycemic Profile in Adults: A Systematic Review of Interventional Studies

**DOI:** 10.1900/RDS.2022.18.10

**Published:** 2022-03-31

**Authors:** Demetrios Bitsanis, Konstantinos Giannakou, Elena Hadjimbei, Stavri Chrysostomou

**Affiliations:** 1 Department of Life Sciences. School of Sciences. European University Cyprus. Nicosia. Cyprus,; 2 Department of Health Sciences. School of Sciences. European University Cyprus. Nicosia. Cyprus.

**Keywords:** early time-restricted feeding, diabetes, glycemic profile, glucose, insulin sensitivity, systematic review

## Abstract

**BACKGROUND:**

Early time-restricted feeding (eTRF) is a new dietary strategy, involving extended fasting (>14h) from midafternoon onwards with or without calorie restriction. Most of the published studies indicate controversial effects on several glycemic markers.

**AIM:**

To evaluate the effect of non-calorie restricted eTRF on the glycemic profile of adults.

**METHOD:**

this systematic review was designed according to PRISMA guidelines. Pubmed/ Medline, the Cochrane library and EBSCO electronic databases were systematically searched for eligible clinical trials. Studies with eTRF or with daily fasting regimens that presented all the characteristics of eTRF were selected and compared with regular diet schedules or delayed time-restricted feeding. Blood glucose and insulin markers were extracted from each study as the main outcome measures.

**RESULTS:**

Five articles including 67 adult subjects in total were selected. The period of intervention varied between 3 days to 5 weeks. Three of the included studies were diet- controlled for weight maintenance, whereas the other two studies allowed for free living. Quality assessment identified two studies of low and three studies of high risk of bias. two studies showed clear positive effects of eTRF on both glucose and insulin markers, including fasting glucose levels, muscle glucose intake, glucose iAUC responses insulin levels, and insulin resistance (p<0.05). Two other studies showed beneficial effects on glucose markers only (fasting glucose, 24h mean glucose levels, and iAUC responses, p<0.05) and the fifth study showed positive effects on insulin markers only (insulin resistance, p<0.05).

**CONCLUSIONS:**

eTRF seems to have positive effects on the glycemic profile mainly in healthy individuals with normal BMI. However, other factors should also be taken into account to address overweight, obese, and prediabetic individuals. Further research is required to clarify better the effectiveness of eTRF among individuals with different characteristics.

## 1 Introduction

The prevalence of obesity and type 2 diabetes Sail (T2D) continues to rise. ‘Diabesity’ is a new term IgUl referring to the co-existence of obesity and T2D and has been predicted to become the biggest epidemic in human history [1-3]. Worldwide, the prevalence of diabetes has increased dramatically during the past decades [3-5], highlighting a strong healthcare and financial burden [6-9]. The strong increase in T2D and other associated metabolic disorders mainly occurs in industrialized countries where excess energy consumption and unhealthy eating patterns are prevalent [10,11]. The Western type of diet and lifestyle which have prevailed over the last decades may have contributed to this development, and therefore health professionals are seeking new cost-effective and easy- to-follow dietary strategies to reestablish optimal glycemic control [12-16].

So far, most of the dietary guidelines for the treatment and prevention of overweight, obesity, and T2D have focused on reducing energy intake and improving diet quality [17]. However, long-term adherence to current guidelines has met with limited success [18]. It seems that the timing of daily food intake is not taken into consideration.

Irregular daily eating patterns have been shown to have adverse effects on circadian biology regardless of the meal size and macronutrient composition [19]. Alterations in Circadian eating patterns are one of the behavioral risk factors leading to obesity and other metabolic diseases [20]. Previous studies found associations between homeostasis and the circadian clock at the behavioral, physiological, and molecular levels, underling that the timing of food intake plays a significant role in the development of obesity and other associated diseases [20]. Therefore, resetting the circadian clock by circadian energy restriction may be useful as a new approach for the prevention of chronic metabolic diseases such as T2D [21-23].

Nowadays, dietary strategies show that focusing on the timing of eating and duration of fasting, known as chrono-nutrition, rather than the type, quality, or quantity of food, has been associated with improved metabolic health outcomes independent of weight loss. In particular, time-restricted feeding (TRF), in which daily food intake is restricted to 8-10 hours, showed beneficial effects on weight loss, glycemic markers, and many other health factors [24,25]. Recently, other TRF subclasses such as early time-restricted feeding (eTRF; tantamount to eating dinner in the mid afternoon), which is better aligned with circadian rhythms [26-31], has gained great popularity. In eTRF, the majority of food intake is shifted to earlier daytimes, with breakfast being served at 7-8am in the morning [29,32], after the cortisol peak [32]. According to this procedure, dinner is served at 2-3pm in the noon [29] and no later than 6pm in the afternoon, during the ghrelin and insulin peak [32], followed by fasting for the rest of the day [29]. This is the major difference of eTRF compared to any other TRF subtypes [29,32].

As commonly known, insulin sensitivity is higher in the morning and declines in the evening [33-36], which is an advantage of eTRF in which the majority of daily energy is consumed earlier in the day before the period when insulin sensitivity is reduced [37]. Notably, previous studies on eTRF demonstrated improved beta-cell function in people with prediabetes [20] and improved glucose levels in individuals with obesity.

To the best of our knowledge, previous studies aiming to evaluate the actual effects of eTRF on glycemic profile of adults are scarce. However, it is impossible to reveal the real effects of this regimen based on these limited data because of the existence of other confounding factors. Most recent systematic reviews and meta-analyses, which have tried to examine the effect of TRF dietary patterns on glycemic markers, have indicated reduced fasting glucose and HOMA-IR in association with a reduced body weight [28,38-40]. However, all previous systematic reviews and metaanalyses included studies with calorie-restricted diets, unspecified meal timing or delayed morning TRF diets (dTRF), and individuals on oral hypoglycemic agents, which may mask the effects of eTRF *per se*.

Recently, health professionals have been trying to develop new dietary strategies for patients with diabetes which should be associated with long-term adherence and improved outcomes, thus limiting the necessity of medication therapy. To the best of our knowledge, there is no previous systematic review examining the effect of eTRF without calorie restriction on the glycemic profile of adults. Therefore, the aim of this systematic review was to examine whether eTRF without caloric restriction improves the glycemic profile of adults.

## 2 Methods

This is a systematic review. The Preferred Reporting Items for Systematic Reviews and Meta-Analyses (PRISMA) guidelines were followed for reporting this study [41,42].

### 2.1 Search strategy

We systematically searched PubMed/MEDLINE, the Cochrane Library, and EBSCO for randomized controlled trials, non-randomized controlled trials, or clinical trials published in the English language that examined the association of eTRF and glycemic profile. The search was conducted independently by two researchers (DB, SC). Discrepancies were resolved by consensus discussion with a third investigator (KG). The search strategy included medical subject heading (MESH) terms or a combination of keywords and MESH terms, as shown in **Table 1** (in the Appendix). We also performed a manual search of the references in the articles retrieved.

**Table 1.** The demographics and baseline characteristics of the eTRF clinical trials

Study year, country	Sample size (n)	Participants	Trial length	Design	Intervention diet and mealtime	Changes in glycemic markers				
						Body weight	Glucose	Insulin	Insulin resistance/sulinogenic index	Main finding
Jones et al.(2020), UK	16	Healthy lean young adult men. Age: 23.0 ± 1.0 years BMI: 24 ± 0.6 kg/ m^2^	2 weeks	Non-RCT, unspecified design, freeliving	eTRF: ad libitum diet/d within 8h (8am- 4pm) CON:CR group matched for nutrient composition (45%CHO:35%FAT:20%P) and energy intake after 2wkof eTRF 1-week baseline period: all	a)73.4±2.97 (before) to 72.36±3.00 (after) 2weeks of eTRF (p<0.05) b) CON:CR vs. eTRF, between group difference (95% CI: body weight -0.20(-1.14, 0.73)), p>0.05	a) Fasting glucose: 4.03±0.08 (before) vs. 4.08±0.17 (after) eTRF, p>0.05 b)Circulating glucose in eTRF, between group difference (95% CI: 93(11, 176) mmol·l-1·180min), main interaction effect p<0.05; n^2^_p_=0.29 c) Muscle glucose uptake in eTRF, [Between group difference (95% CI: 4266(261, 8270) μmol·min-1· Kg-1·180min, p<0.05; n^2^_p_=0.31]	a)Fasting insulin levels in eTRF vs. CON:CR, between group difference (95% CI: 29(8, 49) pmol.l-1), main interaction effect p<0.05; n^2^_p_=0.39 b)Postprandial insulin in eTRF, between group difference (95% CI: 9697(248, 19,146)pmol·l- 1·180min), main interaction effect p<0.05; n^2^_p_=0.26	a)M-ISI in eTRF, between group difference (95% CI: 1.89(0.18, 3.60); intervention group x pre-post trial interaction effect p<0.05; n^2^_p_=0.29	Major effect on insulin sensitivity and skeletal muscle glucose uptake
Jamshed et al. (2019), USA	11	Obese healthy adults (7M, 4F premenopausal). Age: 32 ± 7 years BMI: 30.1 ± 2.7 kg/m^2^	4 days	RCT crossover diet controlled, washout period: ~1mo	eTRF: 3 meals/d within 6h (8am-2pm) Control: 3 meals/d within 12h (8am- 8pm) matched for nutrient composition 50%CHO:35% FAT:15%P) and energy-for-weight maintenance diet	N/A	a) ↓fasting glucose in the morning by 2.0±1.0 mg/dl, p<0.05 b) ↓postprandial glucose after breakfast by 3.0±1.0mg/dl, but NS for p=0.05 c) ↓24-h mean glucose levels by 4.0±1.0mg/dl (by CGM), p<0.0005 d) ↓glycemic excursion by MAGE by 12.0±3.0 mg/dl, p<0.005	a) ↓fasting insulin in the morning by 2,9±0.4mU/l, p<0.0001 b) ↑fasting insulin in the evening by 4.5±1.6mU/l, p<0.02	a) ↓fasting HOMAIR in the morning by 0.73±0.11, p<0.0001, b) ↑HOMA-IR in the evening by 1.09±0.43, p<0.05	Major effect on Glucose Inconsistent effect on insulin and HOMA-IR
Hutchison et al. (2019), Australia	15	Obese older male adults at risk of type 2 diabetes. Age: 55.0 ± 3.0y BMI: 33.9 ± 0.8 kg/m^2^	1 week	RCT crossover diet uncontrolled, washout period: 2 weeks	eTRF: ad libitum diet/d within 9h (8am-5pm) TRFd:9h (12-9pm) 1wk baseline period: all	a)↓Body weight (kg) on7d vs. 0d, p<0.001 (data N/A), but NS between groups (-0.8±0.3; eTRF: -1.3±0.2; TRFd: -0.8±0.2)	a) Fasting glucose, mmol/l, (5.8±0.1 at 0d vs. 5.7±0.1), p>0.05 b)↓fasting glucose by CGM at 7d vs. 0d, p<0.05, but not between treatments, p>0.05 (data N/A) c) ↓glucose iAUC responses to 3-hour meal test by ~36% (-1.6±0.4mmol/ l/h), p<0.005 d) Mean 24-h glucose concentrations and excursions measured by MAGE, CONGA, MODD (data N/A), and postprandial glucose by CGM (data N/A), p>0.05	a)Fasting insulin (mIU/l) at 7d (840±105) vs. 0d (952±108), p>0.05 b) Postprandial insulin, mIU/ lxh our, p>0.05 (404±86 at 0d vs. 328±79 at 7d)		No effect on Insulin Minor effect on glucose
Sutton et al. (2018), USA	8	Prediabetic obese older adult men. Age: 56±9 yr. BMI: 32.2±4.4 kg/ m^2^	5 weeks	RCT crossover diet controlled, washout period: ~7 weeks	eTRF: 3 meals/d within 6h (8am-2pm) Control:3 meals/d within 12h (8am-8pm) 5 days rotary menu in both arms (50% CHO:35%FAT:15%P) and in eTRF calorie adjusted routinely for weight maintenance	a) Body weight, kg, (-1.4±1.3 vs. -1.0±1.1;Δ= -0.5±0.3), p>0.05	a) Fasting glucose (Δ= -2.0±2.0 mg/ dl), p>0.05 b) Mean glucose levels (Δ=5.0±5.0mg/dl), p>0.05	a) ↓Fasting insulin on OGTT by 3.4±1.6mU/l, p=0.05 b)↓Mean and peak insulin values by 26.0±9.0 mU/l and 35.0±13.0 mU/l respectively, p<0.02	a) ↓Insulin resistance by 36.0±10.0 U/mg, p<0.01 b) ↓insulinogenic index on OGTT by 14.0±7.0 U/m, p=0.05	No effect on Glucose Moderate effect on insulin and insulin resistance
Nas et al. (2017), USA	17	Healthy young adults (8M, 9F). Age: 24.6 ± 3.3 years BMI: 23.7 ± 4.6 kg/m^2^	3 days	RCT crossover diet controlled, washout period: 1 days	Skipping dinner (eTRF): 2 meals/d within 6h (7am-1pm) Control:3 meals/d within 12h (7am-7pm) Skipping breakfast (TRFd): 2 meals/d within 6h (1-7pm) Isocaloric diet: (55% CHO:30% FAT:15%P)	N/A	a)Fasting (data N/A) and 24-h glucose (mg/ dl) by CGM (2425±131 in TRFd vs. 2374±165 in eTRF, p>0.05 b) Glucose variations by MAGE (3.65±1.52 in TRFd vs. 3.28±1.75 in eTRF), p>0.05 c) ↓Postprandial glucose (mg/ dl x 2h), eTRF (62.0±40.0) vs. TRFd (114±41.0), p<0.01	a) 24-h insulin secretion (μg/d, 86.0±40.0 in TRFd vs. 75.0±42.0 in eTRF, p>0.05) b)Postprandial insulin (μU/ ml x 2h), eTRF (144±74) vs. TRFd (211±74), p<0.001	a) Fasting HOMAIR (2.07±0.91 in TRFd vs. 1.96±1.05 in eTRF), p>0.05 b) Postprandial HOMApp in eTRF (27.0±23.0) vs. TRFd (59.0±44.0), p<0.01	Minor effect on glycemic markers

**Legend**: CGM - continuous glucose monitoring, CHO – carbohydrate, CON:CR - control: caloric restriction, CONGA - continuous overall net glycemic action, eTRF - early time-restricted feeding, HOMApp - postprandial homeostasis model assessment (of insulin resistance), insulinogenic index - marker of β-cell responsiveness, calculated as the change in insulin divided by the change in glucose during the first 30min, M - male, F - female, MAGE - mean amplitude of glycemic excursions, M-ISI - Matsuda insulin sensitivity index, MODD - mean of daily differences, N/A -not available, non-RCT - non-randomized clinical trial, NS - non-significant, OGTT - oral glucose tolerance test, P - protein, RCT - randomized clinical trial, TRFd - delayed tine-restricted feeding.

### 2.2 Eligibility criteria

The Participants, Investigations, Comparisons, Outcome (PICO) model was employed to define the research question, as shown below [42-44]:

1. Participants: subjects with normal body weight, overweight, or obese (- with or without metabolic and cardiovascular disorders) of any age, sex, ethnicity, and geographical distribution.
2. Interventions: eTRF or daily fasting regimens

that present all the characteristics of eTRF; fasting ≥14h with the first meal served as soon as the subjects are awake (breakfast from 6:00 to 8:30a.m.) and the last meal in the early afternoon (dinner between 13:00-17:00 p.m.). There are no restrictions on the number of meals within the restricted feeding window and no restrictions on calorie intake. However, during the fasting period, no food or beverages containing calories and caffeine can be consumed.

1. Comparisons: standard 24-h daily food intake schedule or other chrono-nutritional patterns (TRF/IF), such as delayed time-restricted feeding (TRFd).
2. Outcomes: blood glucose levels (mg/dl), insulin levels (mU/l), HOMA-IR, or Matsuda insulin sensitivity index (M-ISI).
3. Study design: randomized controlled trials, non- randomized controlled trials, or clinical trials. Exclusion: review articles, animal studies, clinical trials in pregnant or lactating women or infants/children, eTRF or fasting regiments resembling eTRF with caloric restriction, IF clinical trials with antioxidant supplements or dietary supplements that promote body weight reduction or regulate metabolism or oral hypoglycemic agents, and studies on other TRF/ IF dietary patterns.

### 2.3 Data extraction and quality assessment

Data extraction was performed independently following a standardized method by two researchers (DB and SC), while discrepancies were resolved by a third author (KG). The data extracted per study included:

- Author name
-Year of study
-Geographic location
-Age, sex, nationality, and number of participants and their health status
-Study design
-Diet, chrono-nutrition (meal timing)
-Duration of the intervention

The methodological quality of the studies included was assessed by using the Jadad scale [45], which is based on predefined criteria: randomization, double blindness, and withdrawals and dropouts. The final score ranged from 0 to 5 points and was considered “high quality” with a Jadad score ≥3 and “low” quality with a Jadad score ≥2 [45].

## 3 Results

### 3.1 Description of studies

**Figure 1.**
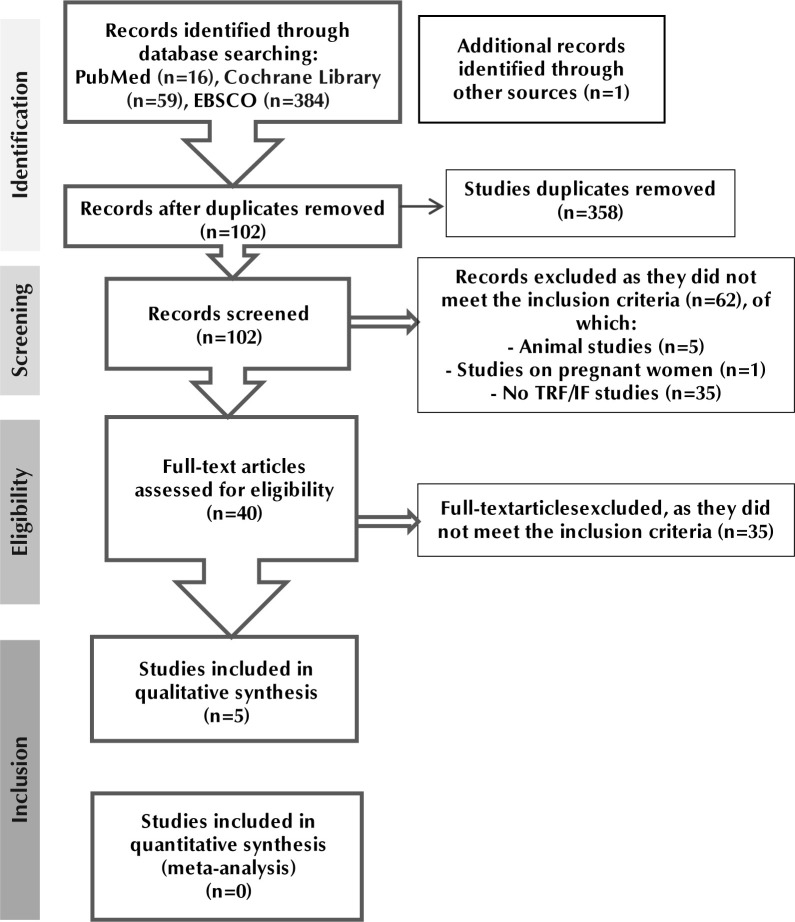
PRISMA flow diagram for the selection of studies of this systematic review. The search strategy was performed based on the PRISMA guidelines.

The initial electronic search identified 460 articles, one of which derived from additional sources. After removing duplicates, 102 records were screened for eligibility, from which 40 full-text publications were retrieved for later evaluation. Among these, 35 articles were excluded because they did not meet the inclusion criteria. A total of five (n=5) clinical trials were considered eligible for inclusion in the current systematic review [46-50]. The flow diagram of the studies included is presented in (**Figure 1**).

### 3.2 Characteristics of the studies included

A total of sixty-seven (n=67) participants were included in this systematic review, fifty-four of which (n=54) were men and the rest (n=13) were women. The baseline characteristics of the study subjects are shown in Table 1. The studies by Nas et al. (2017), Sutton et al. (2018), and Jamshed et al. (2019) were diet-controlled RTCs [47-49], whereas the remaining two studies by Hutchison et al. (2019) and Jones et al. (2020) were diet-uncontrolled (free living) [46,50]. The diet-controlled RCTs (n=3), composed of three [47,49] or two meals daily [48], were designed to meet weight-maintenance energy requirements to ensure that any changes in metabolic indices were not related to weight loss. In the uncontrolled trials (free living) [46,47], the participants were advised to maintain their usual diet within the specified restricted feeding period throughout the intervention. The duration of eTRF trials varied from 3days to 5weeks. Similarly, the wash out period also differed (1day to 7weeks) between interventions with crossover design [46-49]. In the clinical trials, the participants were assigned to different daily fasting-to-feeding regimens (hours): 15:9 [46], 16:8 [50], and 18:6 [47-49].

### 3.3 Effect of eTRF on blood glucose levels

The study by Hutchison et al. (2019) reported a reduction in fasting glucose levels in the eTRF group (p<0.05) compared with baseline [46]. In the same study, both eTRF and TRFd reduced glucose iAUC responses to a 3-h meal test by ~36% (p<0.005) [46].

Moreover, the study by Jamshed et al. (2019) indicated that fasting glucose levels were decreased (p<0.05) in the morning in the eTRF group compared to the control group [47]. The same study, showed that a 4d-eTRF decreased mean 24-h glucose levels (p<0.0005) and 24-h glycemic excursions (p<0.005) in the intervention group compared with the control group [47]. Likewise, the study by Jones et al. (2020) showed that 2wk of eTRF reduced circulating glucose 3hours after ingesting a carbohydrate (CHO) and protein drink (p<0.05) compared with baseline, and increased muscle glucose uptake (p<0.05) compared with the control group [50]. Moreover, in the study by Nas et al. (2017), although 24-h glycemic was similar between the interventions, skipping dinner (resembling eTRF) reduced postprandial glucose iAUCs after lunch (p<0.001) compared to the skipping breakfast (resembling TRFd) intervention [48]. The findings of the studies included regarding the effect of eTRF on blood glucose levels are shown in **Table 1**.

### 3.4 Effect of eTRF on insulin levels and insulin resistance/sensitivity

The study by Jamshed et al. (2019) showed that fasting insulin (p<0.02) and HOMA-IR (p<0.0001) were significantly decreased in the morning and increased in the evening compared to the control group [47]. The study by Sutton et al. (2018) showed that 5wk of eTRF significantly reduced mean and peak insulin levels (p<0.01) [49]. Also, the same study showed that 5wk of eTRF reduced insulin resistance (p<0.01), as measured by the 3-hour incremental AUC ratio, compared with the control group [49]. Moreover, the study by Jones et al. (2020) showed that fasting insulin levels were significantly higher in the eTRF than in the control group (p<0.02) after 2weeks of intervention [50]. The same study found that 2wk of eTRF reduced insulin levels across the entire postprandial period (p<0.05) and significantly improved M-ISI in response to consumption of a liquid test meal (p<0.05) [50].

The study by Nas et al. (2017) showed that postprandial insulin was significantly lower in the skipping dinner group (resembling eTRF) compared with the skipping breakfast group (resembling TRFd) (p<0.01) [48]. Also, the same study showed that skipping dinner (resembling eTRF) reduced postprandial HOMApp after lunch compared with the skipping breakfast group (resembling TRFd) (p<0.01) [48]. The findings of the studies included regarding the effect of eTRF on insulin levels and insulin resistance/ sensitivity are shown in Table 1.

### 3.5 Quality assessment

The quality assessment of the studies included revealed that two RCTs (n=2) with Jadad score=3 validity criteria were of high quality [47,49], while the remaining three clinical studies (n=3) with Jadad score ≥2 were of low quality [46,48,50]. The results of the quality assessment of the studies included are shown in **Table 2**.

**Table 2.** Quality assessment of four clinical trials based on the Jadad scale

Clinical trial	Randomization		Double blindness		Reported withdrawals and dropouts	Jadad score (max =5)	Risk of bias
	Reported	Appropriately	Reported	Appropriately			
**Jones et al., 2020**	00	00	00	00	111	1/51/5	High
**Hutchison et al., 2019**	1	0	0	0	1	2/5	High
**Jamshed et al., 2019**	1	1	0	0	1	3/5	Low
**Sutton et al., 2018**	1	1	0	0	1	3/5	Low
**Nas et al., 2017**	1	1	0	0	0	2/5	High

Jadad score≥3; high methodological quality and Jadad score ≥2; low methodological quality

## 4.Discussion

This is the first systematic review to examine the effects of eTRF without caloric restriction on the glycemic profile of adults. Based on the results, 2 out of 5 studies [48,50] showed a clear positive effect of eTRF on glycemic profile, for both glucose and insulin markers. Notably, both studies included healthy individuals of normal mean body mass index (BMI). The other 2 studies [46,49] indicated positive effects, but not for all glycemic markers.

Sutton et al. (2018) showed positive effects for insulin markers [49], whereas the study by Hutchison et al. showed positive effects for glucose markers [46]. Additionally, the study by Jamshed et al. (2019) showed clear effects through glucose markers, but the results regarding insulin markers were conflicting [47]. Overall, the current findings support the conclusion that all studies demonstrated a beneficial effect of eTRF on glycemic profile by at least one glycemic index (glucose or insulin). Therefore, this new dietary pattern could be a promising strategy for preventing and/or treating chronic metabolic diseases such as T2D.

In the current systematic review, the period of intervention, type of intervention (eTRF controlled vs. eTRF free living), and sample characteristics differed among the studies included; this is probably the reason for the conflicting results. However, it is necessary to identify these factors clearly to enable health professionals to apply eTRF as a new dietary strategy for improving glycemic control.

Two of the studies [46,49] included populations with similar characteristics (overweight and obese, high risk of diabetes participants), but yielded different results. In particular, the short-term study by Hutchison et al. (1 week) showed positive improvements on glucose (decrease glucose iAUC responses), but no effect on insulin markers [46]. On the other hand, the long-term study by Sutton et al. (5weeks) showed no improvement on glucose markers, but a significant decrease in insulin resistance [49]. Therefore, it could be assumed that among overweight, obese, and prediabetic individuals the duration or type of intervention (calorie- vs. non calorie-restricted) may hold the key to potential effectiveness. Short-term interventions are more likely to affect glucose markers, whereas longer interventions are more likely to affect insulin markers.

The different types of intervention (diet-controlled vs. free living) did not seem to affect the results since both studies demonstrated positive effects on glycemic profile, although on different markers.

Notably, two other studies of the same type of intervention (i.e. diet-controlled) and similar periods of intervention but different study population yielded different findings. In particular, the study by Nas et al. (2017) of a 3-day intervention included healthy individuals of normal mean BMI and showed a significant decrease in postprandial glucose and insulin and HOMApp levels in the eTRF group compared with the dTRF group [48] , whereas the study by Jamshed et al. (2019) of similar duration (4-day intervention) but different study population (healthy and obese participants of mean BMI=30.1kg/ m^2^) showed improvements in fasting glucose and 24-h glucose levels, but the findings on insulin markers were conflicting (fasting insulin and HOMA-IR were decreased in the morning, but increased in the evening) [47]. However, based on these two studies [47,48], it seems that the effects of eTRF depend on population characteristics rather than the period of intervention, and it is probably more effective among healthy people with normal mean BMI than overweight or obese people. In the case of overweight or obese people, other factors should be also taken into consideration such as the induction of weight loss through energy-restricted dietary models. Nevertheless, the free-living study of a 2-week intervention among healthy individuals with normal mean BMI also yielded positive results with decreased circulating glucose and post-prandial insulin and increased muscle glucose uptake and insulin M-ISI [50].

Overall, it seems that the effectiveness of eTRF on the glycemic profile of adults mainly depends on individual characteristics. It seems that eTRF is more likely to provide positive effects among healthy individuals of normal BMI than among overweight, obese, or pre-diabetic individuals. In the case of overweight, obese, and prediabetic adults, more studies are required to clarify the ideal intervention period and type of intervention (diet-controlled vs. free living vs. caloric restriction) for optimal glycemic outcomes.

Restricted eating (>16h) alone resulted in a significant caloric reduction and consequent weight loss [39]. Therefore, most of the included trials were diet- controlled to ensure that body weight was maintained during the intervention period and to imply that the effect throughout the interventions was a result of the eTRF regimen only. However, our findings indicate that controlling the caloric intake of a diet so as to avoid changes in weight is not necessarily an important contributor to a positive outcome since studies that were uncontrolled [46,50] also showed positive effects. Notably, the uncontrolled study by Jones et al. [50], showed positive effects through multiple markers. Therefore, it could be assumed that other factors rather than weight loss may contribute to the effectiveness of eTRF on glycemic profile. A possible explanation could be that the timing of feeding and subsequent resetting of the circadian “clock” were caused by the eTRF regimen. The circadian “clock” resets physiological and metabolic procedures such as cardiovascular and renal activity as well as the activity of the endocrine system and human metabolism [51,52]. The central circadian “clock” is located in the brain, and is composed of multiple oscillators aiming to synchronize circadian rhythms [52]. However, this “clock” requires resetting each day to the external light-dark cycle to prevent its drifting out of phase, because light is a strong synchronizer for the brain clock. A previous study by Halberg showed that eating breakfast only was associated with weight loss, whereas this was not observed when eating the same number of calories as dinner only [53]. Also, the same study showed that the configuration of the circadian system was different based on whether eating took place in the morning or evening under the same conditions. Therefore, feeding time seems to play an important role for optimal metabolic outcomes.

Hence, our findings do not fully agree with previous research, probably because of differences in study methodologies including intermittent fasting (IF), TRF, eTRF, and dTRF regimens. Particularly, the meta-analysis by Pureza et al. (2021), indicated a significant reduction in fasting glucose levels and HOMA-IR associated with increased weight loss [28]. However, this meta-analysis included studies that were calorie-restricted (skipping breakfast) or of other TRF subtypes (dTRF) which are able to mask the effects of eTRF per se. Nonetheless, other meta-analyses related to TRF regimens found that the beneficial effect on glucose levels was associated with weight loss [34,40]. Moreover, another meta-analysis indicated significant positive effects of IF and TRF on body weight, fasting glucose, and HOMA-IR [38]. However, it would appear that, in all previous meta-analyses, effects on glycemic profile were shown in conjunction with weight loss, and thus it could be assumed that calorie-restricted feeding could reset circadian rhythms independently [20] and may enhance the effectiveness of the TRF intervention on glycemic profile. However, our systematic review indicates that eTRF may also improve glycemic profile without weight loss, but that the size of effectiveness is different and based on other factors such as individual characteristics.

We should acknowledge some limitations of our work. One is that our systematic review included only five clinical studies with a small number of participants. Therefore, many of the effect estimates were associated with large uncertainty, making it difficult to draw generalized conclusions. This may have negatively affected the outcome of this review, and it seems that more research is necessary particularly among overweight and obese individuals. Moreover, there was a high heterogeneity among the study population (healthy individuals, overweight, obese, and pre diabetic). Due to the great diversity in study populations, it was not possible to conduct a meta-analysis. Moreover, the short duration of the studies included in this systematic review (3 days up to 5 weeks) makes interpretation of the results more difficult. Finally, more than half of the clinical trials were considered to be of low methodological quality.

## 5 Conclusions

The eTRF dietary strategy is a new feeding pattern based on the timing of eating which emphasizes circadian reprogramming as an entraining signal to reset metabolic cycles including glucose metabolism [54]. This systematic review showed that TRF may positively affect glycemic profile under specific circumstances. It seems that healthy people with normal BMI are more likely to benefit regardless of the period of intervention or type of diet (controlled vs. free living), but in the case of overweight, obese, and prediabetic individuals the effectiveness is likely to be decreased probably due to other factors such as the period and/or type of intervention.

It is probable that eTRF with caloric restriction inducing weight loss could be more effective among overweight/obese and pre diabetic individuals. As data are lacking on the topic, it is expected that our results can help to shed light on the possible effects of eTRF on the glycemic profile of adults and guide future interventional studies. More high-quality studies of populations with similar characteristics examining the effects of the circadian rhythm-reinforcing eTRF compared with other chrono-nutrition regimens are required to explore better the effectiveness of eTRF on glycemic profile.

## Abbreviations

BMI: body mass index

CGM: continuous glucose monitoring

CHO: carbohydrate

CON:CR control:caloric restriction

CONGA: continuous overall net glycemic action

eTRF: early time-restricted feeding

HbA1c: glycated hemoglobin A1c

HOMA-IR: homeostasis model assessment of insulin resistance

HOMApp: postprandial homeostasis model assessment (of insulin resistance)

iAUC: incremental surface area below the curve

IF: intermittent fasting

MAGE: mean amplitude of glycemic excursions

M-ISI: matsuda insulin sensitivity index

MODD: mean of daily differences

OGTT: oral glucose tolerance test

P: protein

RCT: randomized controlled trial

TRF: time-restricted feeding

TRFd: delayed time-restricted feeding
